# Comparison of Ground-Based and Satellite-Derived Solar UV Index Levels at Six South African Sites

**DOI:** 10.3390/ijerph14111384

**Published:** 2017-11-14

**Authors:** Jean-Maurice Cadet, Hassan Bencherif, Thierry Portafaix, Kévin Lamy, Katlego Ncongwane, Gerrie J. R. Coetzee, Caradee Y. Wright

**Affiliations:** 1 LACy, Laboratoire de l’Atmosphère et des Cyclones (UMR 8105 CNRS, Université de La Réunion, Météo-France), Saint-Denis de La Réunion 97744, France; hassan.bencherif@univ-reunion.fr (H.B.); thierry.portafaix@univ-reunion.fr (T.P.); kevin.lamy@univ-reunion.fr (K.L.); 2 School of Chemistry and Physics, University of KwaZulu-Natal, Durban 4041, South Africa; 3 South African Weather Service, Private Bag X097, Pretoria 0001, South Africa; katlego.ncongwane@weathersa.co.za (K.N.); gerrie.coetzee@weathersa.co.za (G.J.R.C.); 4 Environment and Health Research Unit, South African Medical Research Council, Pretoria 0001, South Africa; Caradee.Wright@mrc.ac.za; 5 Department of Geography, Geoinformatics and Meteorology, University of Pretoria, Pretoria 0002, South Africa

**Keywords:** solar ultraviolet radiation, UV index, ground-based measurements, satellite-derived data, OMI/AURA, South Africa

## Abstract

South Africa has been measuring the ground-based solar UV index for more than two decades at six sites to raise awareness about the impacts of the solar UV index on human health. This paper is an exploratory study based on comparison with satellite UV index measurements from the OMI/AURA experiment. Relative UV index differences between ground-based and satellite-derived data ranged from 0 to 45% depending on the site and year. Most of time, these differences appear in winter. Some ground-based stations’ data had closer agreement with satellite-derived data. While the ground-based instruments are not intended for long-term trend analysis, they provide UV index information for public awareness instead, with some weak signs suggesting such long-term trends may exist in the ground-based data. The annual cycle, altitude, and latitude effects clearly appear in the UV index data measured in South Africa. This variability must be taken into account for the development of an excess solar UV exposure prevention strategy.

## 1. Introduction

Solar ultraviolet radiation (UVR) is known to have biological effects on ecosystems, plants, animals, and humans [1]. Solar UVR is usually divided into three wavebands [2]: UVA: 315–400 nm; UVB: 280–315 nm; and UVC: 100–280 nm. All UVC, potentially the most dangerous UVR band, is absorbed by ozone and oxygen in the atmosphere and, therefore, does not reach the Earth’s surface, while UVA is weakly absorbed by ozone, and only a fraction of UVB reaches the surface with the majority being absorbed by ozone. The important implications of UVA and UVB on human health are translated via the application of an action spectrum, here, specifically, for erythema (sunburn) which occurs due to excess UVA and UVB exposure [3]. The Ultraviolet Index is a standard unitless measure of UVR used to describe an erythemal dose rate where 1 UV index unit is equivalent to 25 mW∙m^−2^ [4]. The UV index is recommended as a public communication tool to convey solar UVR levels and appropriate sun protection advice [4]. Several countries around the world monitor ground-based solar UVR and/or forecast a predicted UV index reading for the general public on a daily basis.

South Africa, located in the mid-latitudes between 22° S and 35° S, has been measuring ground-based UV index levels since 1990. The network was originally initiated due to concerns regarding the possible adverse health impacts of excess personal solar UVR exposure on the South African population. Environmental monitoring was deemed an important approach to provide observational evidence for the typical levels of solar UV index across the country. These data would help to understand the results of studies showing relatively high UV index patterns [5], as well as high incidence of skin cancers, especially among Caucasian groups [6]. Furthermore, ozone depletion has been an international concern from the late 1970s. Therefore, there was a need for local ground-based UV index data across South Africa to consider health impacts relative to changing stratospheric ozone levels and possible human health impacts. Thus, the network was initiated comprising four sites, initially, with two additional sites being added later.

Several studies were conducted during the 1990s and early 2000s to consider both ground-based and satellite-derived UV index levels at South African sites. Past (1978–1994) and future (1998–2007) UV index trends were assessed using data from the satellite-borne TOMS (Total Ozone Mapping Spectrophotometer) instruments for Johannesburg, Bloemfontein, Durban, Cape Town, and Port Elizabeth [7]. Small increases in the UV index levels were predicted for the early 2000s. The UV index measured with a pyranometer in Durban between 1996 and 1998 showed good agreement with satellite data of annual erythema irradiance curves [8]. While the period was too short to test for long-term trends, a positive anti-correlation was found between UV index and total column ozone.

To date, the full dataset measured by the ground-based UV index network instruments has not been analysed or compared to satellite-derived UV index data for the same sites. A process of data verification or qualification is necessary should these data be used for long-term trend analysis, or for the monitoring of solar UVR exposure risk and possible impacts on human health. Studies have shown the complexities of applying satellite-derived UV index estimates for surface UV index levels, where relative differences between the two UV index measures range from 0 to 20% [9,10,11,12,13,14,15,16,17]. Satellite-derived UV index data is largely affected by spatial and temporal cloud modification effects, among other factors, such as total column ozone, aerosols, albedo, etc. [18], that cannot be accurately incorporated in the algorithm or model-calculated UV index levels.

Given these complexities, the aim of the study reported here is to compare the available ground-based and satellite-derived solar UV indices at six South African sites. Two objectives were identified: (1) to compare ground-based and satellite-derived solar UV index levels at the six sites; and (2) to provide an estimate of the quality of the ground-based solar UV index data. No attempts were made to correct the ground-based solar UV index data; the findings are discussed in terms of the challenges of using ground-based and satellite-derived solar UV index datasets to, in the most accurate manner, determine the surface solar UV index.

For the first time, data and methods of comparison will be presented. Afterward, the result of the comparison will be shown and discussed, before gathering our findings and reporting our conclusion.

## 2. Data and Methods

### 2.1. Ground-Based Solar UVR Data

**Figure 1:**
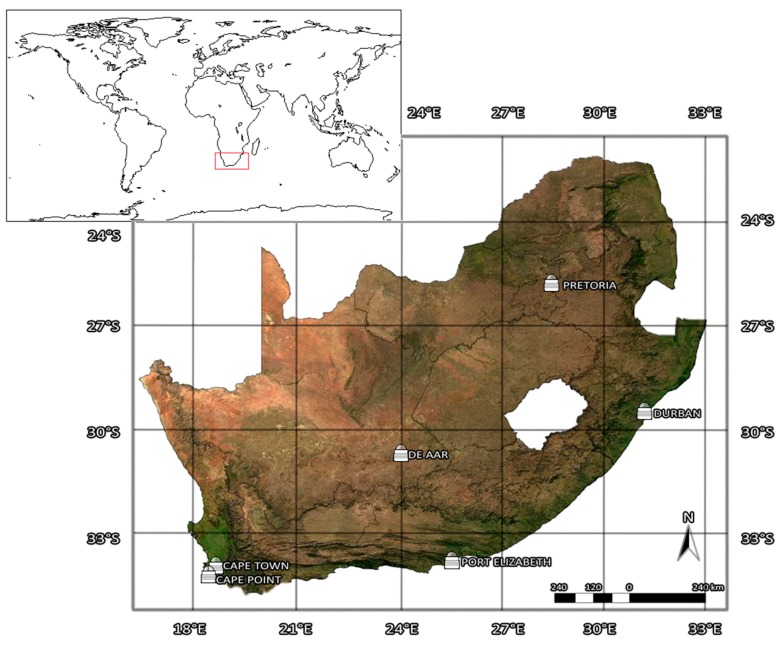
Geographical locations of the six South African Weather Service ground-based solar UVR measurement sites in South Africa.

In 1994, the South African Weather Service (SAWS) implemented a network of three UV biometers at Cape Town, Durban, and Pretoria (Figure 1). The Cape Point station was added to the network in 1997, and two other stations were added later, namely Port Elizabeth station in 2000 and De Aar station in 2002. All geographical information is presented in Table 1.

Solar UVR (280–340 nm) is recorded using a broadband instrument: a model 501 UV-biometer manufactured by Solar Light Pty Ltd. (Glenside, PA, USA). A GaAsP diode, protected by a quartz dome, collects the solar UV radiation, and the electrical intensity is converted into a minimum erythemal dose per hour (MED), where 1 MED is equivalent to 210 J∙m^−2^. Measurements are made hourly to determine a MED/h. For the purpose of this study, MED/h readings from the UV biometers were converted to a UV index using Equation (1). A correction of the spectral response depending on total ozone and solar zenith angle was applied by using a generic table for the model 501 UV biometer to convert the instrument-weighted UVR to the erythemally-weighted UVR [19].

(1) $$UV\;index=UVd\left[MED\cdot h^{-1}\right].\;\frac{210\left[J\cdot m^{-2}\right]\;\cdot \;40\left[m^{2}\cdot W^{-1}\right]}{3600\left[s\right]}$$

where UV index is the hourly UV index and *UVd* is the hourly erythemally-weighted exposure [20].

The accuracy of the UV biometer used is ±5% of the daily total. Taking into consideration the installation, the maintenance, and the temperature control, the uncertainty can reach up to ±8%. The biometer at Cape Point station was calibrated in 2012 at the Meteorological Observatory Service Deutscher Wetterdienst by using the spectrometer SPECTRO 320D NO 15 (Instrument Systems GmbH, Munich, Germany). The calibration was done by using the sun as the source and by comparison of the two instruments.

### 2.2. Satellite-Derived Solar UVR Levels

Launched in late 2004, the Aura satellite is dedicated to measuring ozone, aerosols, and other key gases in the atmosphere. The OMI (Ozone Monitoring Instrument) sensor aboard Aura measures ozone total quantities by analysing the backscattered solar radiation. The erythemally-weighted irradiance (290–400 nm) is computed by using an enhanced version of the TOMS (Total Ozone Mapping Spectrometer) surface UV-B flux algorithm [11,21]. A recent version of OMI datasets taking better consideration of aerosol optical depth and single scattering albedo obtained by modelling and ground-based observations from the AERONET network was used [22]. Data from OMI/Aura for the UV index given at overpass are available on the GIOVANNI platform for download [23].

We applied the ‘all-sky UV index’ data and not the ‘clear-sky UV index’ data in this study, since the ground-based solar UV index data included the effects of clouds, where clouds are known factors influencing ambient solar UVR levels [1]. We did not have cloud data at the ground sites, therefore, we were unable to identify clear sky only days. The OMI data are gridded at 1° × 1° [24]. Satellite data from 2005 to 2015 (i.e., 10 years) overpass time UVI measurements are used in these analyses. The uncertainty is 2 to 5% for small to large solar zenith angles.

### 2.3. Methods and Statistical Analysis

Both datasets were prepared separately and scrutinised for obvious errors. Erroneous night-time UV index values were removed. The ground-based dataset for Durban exhibited a 2-h time shift in instrument timing between 2009 and 2012 and this timing error was corrected. Ground-based data were analysed for diurnal, monthly, and annual trends and satellite-derived data for monthly and annual trends since one value (overpass satellite time) of the UV index was available per day. Both datasets were compared at satellite overpass time (around 1:45 p.m. for South Africa). To only keep clear sky days for the comparison, OMI Lambertian equivalent reflectivity (LER) was used. The days with an LER higher than 10% were removed [25].

A comparison of ground-based and satellite-derived solar UV index values were made in two ways: (1) computation of a daily correlation year-by-year to see if ground-based and satellite data evolve in the same way; and (2) computation of a daily bias year-over-year to provide an estimate of the relative difference between the two datasets.

To compare the ground-based data with satellite data, we calculated the bias, median, standard deviation, and root mean square error (RMSE). Bias is defined as the ground-based observation subtracted by the satellite observation and can be interpreted as the difference between the two datasets. The median separates the data in two equal parts. Standard deviation is defined as the dispersion of the previous bias. RMSE is defined as a measure of the differences between calculated values (here, OMI data) and observed values, i.e., ground-based observations. Lower RMSE values indicate less residual variance.

The equation (Equation (2)) for the R-squared correlation is given below:

(2) $$r=\frac{\sum_{i=1}^{n}\left(S_{GND,i}-\bar{S_{GND}}\right)\left(S_{OMI,i}-\bar{S_{OMI}}\right)}{\sqrt{\left(\sum_{i=1}^{n}{\left(S_{GND,i}-\bar{S_{GND}}\right)}^{2}\right)\left(\sum_{i=1}^{n}{\left(S_{OMI,i}-\bar{S_{OMI}}\right)}^{2}\right)}}$$

where S is the UV index, $\bar{S}$ is mean UV index, *GND* is the ground-based observation, *OMI* is the satellite observation, and *n* the number of observation.

We computed a bias (Equation (3)) and mean absolute percentage error (MAPE) (Equation (4)), a standard deviation (Equation (5)), and an RMSE (Equation (6)) as given below:

(3) $$Bias=\frac{1}{n}\sum_{i=1}^{n}\left(S_{OMI,i}-S_{GND,i}\right)$$

(4) $$MAPE=\frac{1}{n}\sum_{i=1}^{n}\left(100*\frac{\left|S_{OMI,i}-S_{GND,i}\right|}{S_{GND,i}}\right)$$

(5) $$Std=\sqrt{\frac{1}{n-1}\sum_{i=1}^{n}{\left(\left(S_{OMI,i}-S_{GND,i}\right)-Bias\right)}^{2}}$$

(6) $$RMSE=\sqrt{\frac{1}{n-1}\sum_{i=1}^{n}{\left(S_{OMI,i}-S_{GND,i}\right)}^{2}}$$

where *S* is the UV index, *GND* is the ground-based observation, *OMI* is the satellite observation, and *n* is the number of observations. These calculations were made for the full period of comparison, i.e., 2005 to 2015, as well as year-over-year in order to quantify the temporal evolution of change in solar UVR data from ground-based sites and satellite-derived observations.

## 3. Results

### 3.1. Ground-Based Solar UVR Observations

**Figure 2:**
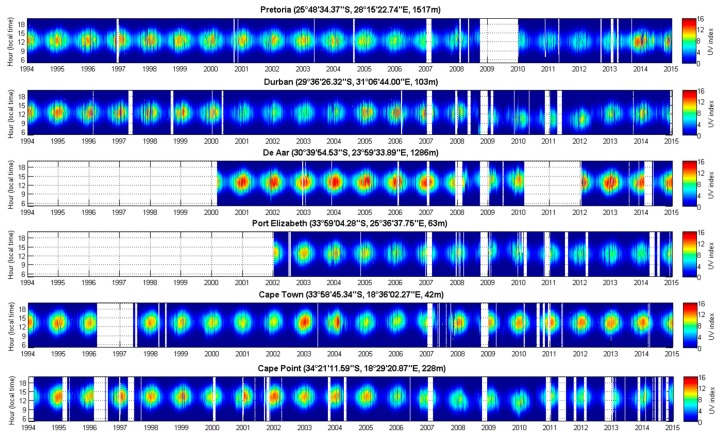
UV index observations as a function of day and hour per day for Cape Point, Cape Town, Durban, De Aar, Port Elisabeth, and Pretoria from 1994 to 2015.

All solar UVR results are given in UV index units. Hourly data from the six ground-based solar UVR measurements sites were analysed for the period 1994–2015 where data were available for each site. Raw data are provided in Figure 2. Four stations initiated observations in 1994, while De Aar and Port Elizabeth observations begun later, in 2000 and 2002, respectively. Several data gaps are also evident, with the longest being at De Aar between 2010 and 2012. The typical diurnal solar UVR pattern is evident with UV index levels increasing during the day, reaching a peak at solar noon and decreasing during the afternoon as solar zenith angles increase again. Annual variability, modulated by the seasonal cycle, is also evident in Figure 2, with summer UV index values regularly exceeding 11 UV index units at De Aar. De Aar presents a clear atmosphere because of its isolated geographical position and its relatively low industrial activity [26]. Similar results on these time series have been found previously [27,28].

**Figure 3:**
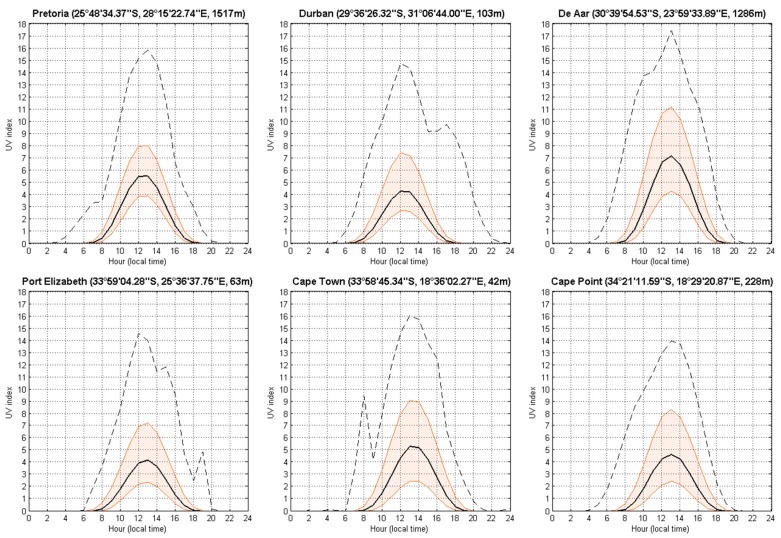
Daily climatological median (black line) of the solar UVI for the six South African sites, for all years of observation (i.e., 1994–2015). Quantile 25 and 75 (filled orange lines) and minimum and maximum (black dash line) are also represented.

The effects of latitude and altitude on solar UV index levels are apparent in Figure 3 where South African sites situated closer to the equator and at higher altitude tend to experience higher mean solar UV index levels, i.e., De Aar (mean: 8) and Pretoria (mean: 6) compared to other sites.

**Figure 4:**
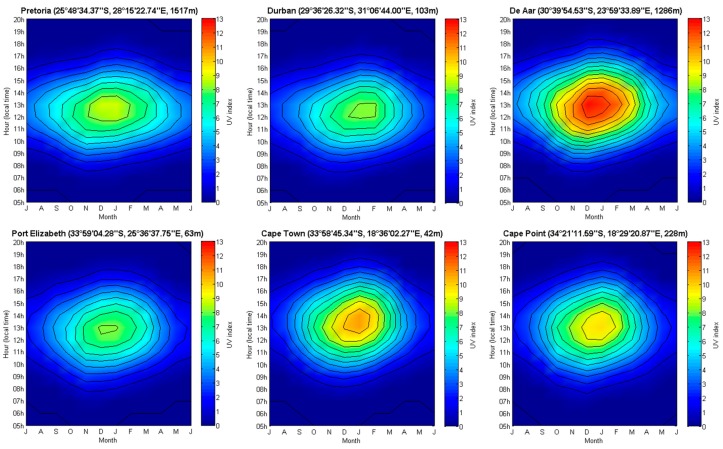
The monthly climatological median ground-based solar UV index levels for all six South African sites for all available years between 1994 and 2015 shown as a function of time of day and month of the year. For seasons to be shown more easily, the plots show the first month on the *X*-axis as July through to December, followed by January through to June. Therefore, summer months are in the middle of the plot.

Figure 4 shows the monthly climatological median ground-based solar UV index levels for all six South African sites for all available years between 1994 and 2015 shown as a function of time of day and month in the year. Annual variability and change in UV index levels by season are emphasized. Solar UV index levels are the highest during the austral summer and lowest during the winter. It is apparent that the hour of maximum intensity (i.e., daily peak UV index) depends on the longitude of the site. Cape Town had the latest mean hour of maximum UV index followed by De Aar, Port Elizabeth, Pretoria, and Durban.

**Figure 5:**
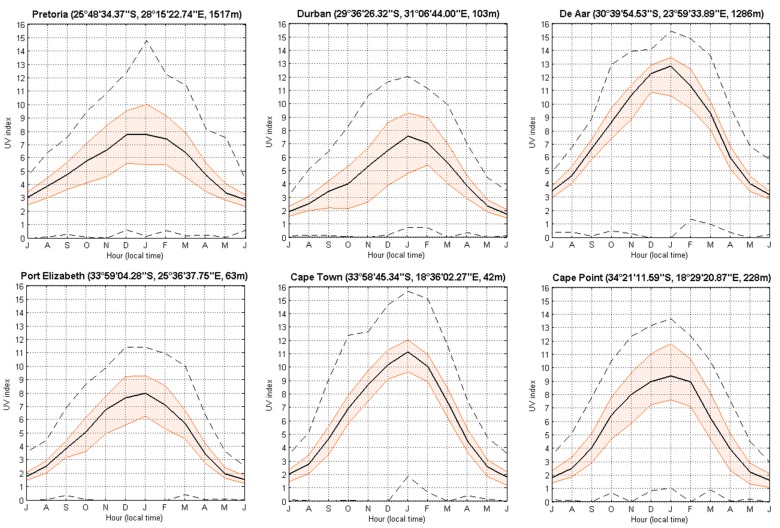
Monthly climatological median (black line) of the solar UVI for the six South African sites at satellite overpass time, for all years of observation (i.e., 1994–2015). Quantile 25 and 75 (filled orange lines) and minimum and maximum (black dash line) are also represented.

Since the satellite-derived solar UVR data were only available for the period of 2005 to 2015, the ground-based solar UVR dataset was reduced, for comparison purposes and further analysis (see Section 3.3 below), to only include the UV index at satellite overpass time readings from 2005 to 2015 (Figure 5).

If we do not consider De Aar, which is the second highest and the clearest site, Cape Town shows the more exposed site to UVR around noon. Cape Town and Cape Point show similar UVR levels; they are two geographically-close sites (about 50 km apart).

### 3.2. Satellite-Derived Solar UVR Measurements

**Figure 6:**
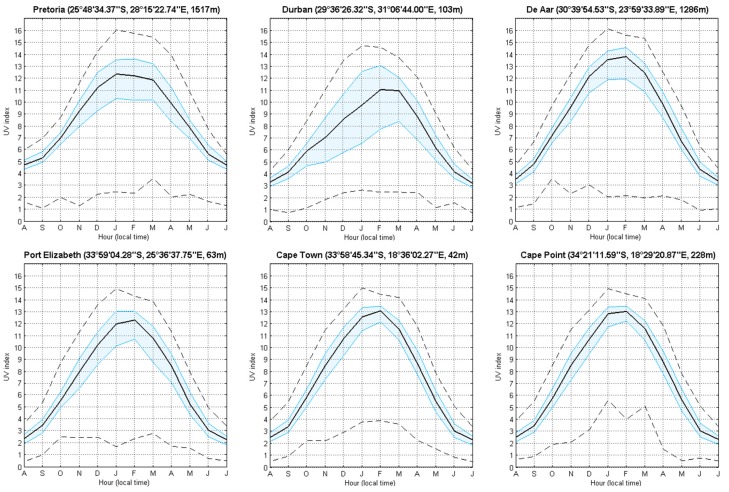
Monthly climatological median (black line) of solar UVI for the six South African sites at satellite overpass time, for all years of observation (i.e., 1994–2015). Quantile 25 and 75 (filled blue lines) and minimum and maximum (black dash line) are also represented.

Satellite-derived UV index values at overpass time above all sites were similar with maximum UV index levels of ~15 and low levels of ~2. For all sites, scatter about the mean is mostly tight with the most scatter evident at the coastal site of Durban. The yearly UV index median was highest at De Aar, followed by Pretoria and lowest at Port Elizabeth (see Figure 6). The UV index profiles at Cape Town and Cape Point are similar because of the proximity of the two measurements’ sites, as they are separated by ~50 km.

### 3.3. Comparison of Ground-Based and Satellite-Derived Solar UVR Data

**Figure 7:**
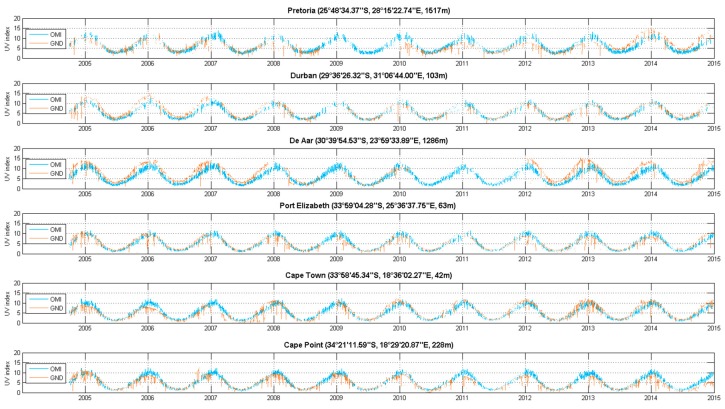
Time series comparing ground-based versus satellite-derived solar UV index data for 2005 to 2015 at satellite overpass time (the comparison was performed at the satellite overpass time and only clear sky days are selected with LER).

Ground-based UV index measurements at satellite overpass time were compared with those derived from the satellite OMI measurements for the six South African sites and for the period 2005–2015 (Figure 7). A similarity in the timing patterns of seasonal peaks is apparent in the two datasets.

A visual comparison shows large differences for certain periods of time between the instruments. For example, at Cape Town in 2007, a new logger was used. At De Aar, in February 2007 the logger was changed because of lightning damage. At Pretoria, in 2013, the amplitude increases suddenly because of the roof was painted silver, although the sinusoidal pattern is preserved.

**Figure 8:**
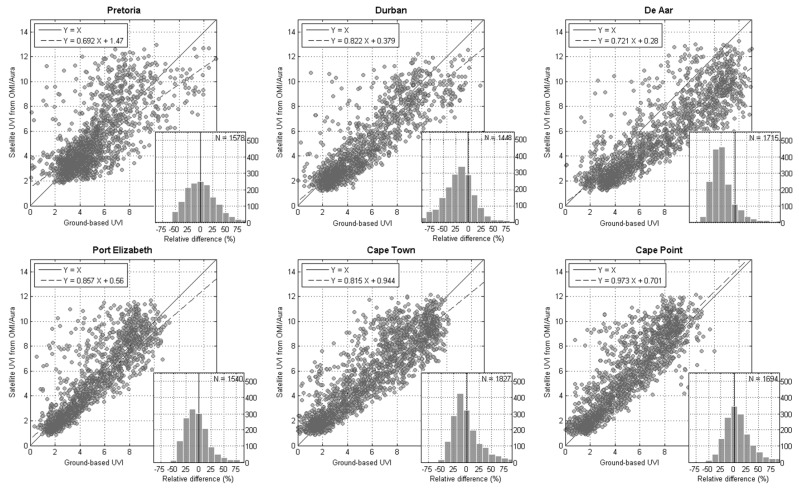
Correlation between the ground-based UV index and satellite-derived UV index data for 2005 to 2015 at the six South African sites at satellite overpass time. The six inserted histograms show the relative difference between the two datasets by the number of observations for each respective site (the comparison was performed at the satellite overpass time and only clear sky days are selected with LER).

**Figure 9:**
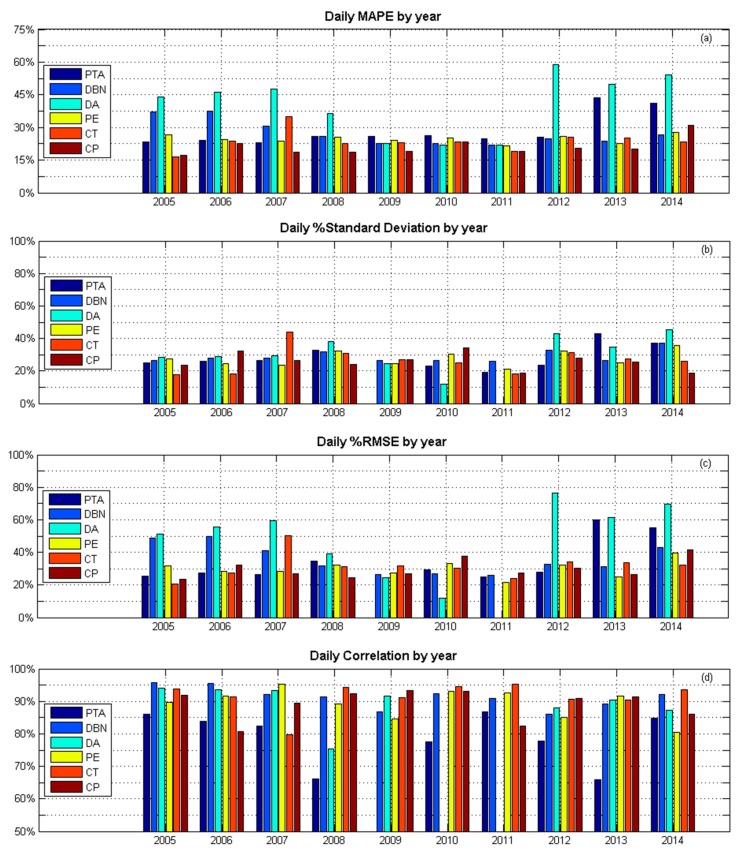
Year-on-year validation statistics for daily clear sky satellite overpass time UV index values at each of the six sites: Cape Point (CP), Cape Town (CT), Durban (DBN), De Aar (DA), Port Elizabeth (PE), and Pretoria (PTA), where (**a**) shows the daily MAPE by year; (**b**) the daily percentage standard deviation by year; (**c**) daily percentage RMSE; and (**d**) daily percentage correlation by year.

Correlation analysis confirmed a positive correlation between the two datasets (Figure 8) where R^2^ values ranged from a moderate correlation co-efficient of 0.71 for Pretoria, to a strong correlation co-efficient of 0.88 for Durban (Table 2). These high correlations are due to the dominating annual course of the two datasets. The MAPE between the two datasets tended to be between 22% and 28%, 46% for De Aar. Hence, the ground-based UV index observations are in good agreement with satellite measurement, except for De Aar where ground-based observations are higher (Figure 9).

Variability is evident in the year-over-year analyses of MAPE, standard deviation, and RMSE. The yearly MAPE range from 15 to 30%, except for De Aar, where a 60% MAPE can be reached. A relatively constant bias is observed at Port Elizabeth around 15%. At the Pretoria station, the bias decreases significantly in 2013 because of a change of surface albedo (this effect is considered to have reduced since 2015 with fading of the roof paint). The daily percentage RMSE values by year also varied across years and sites, but generally stayed within the 30% range (Figure 9c). The largest change in correlation percentage (25%) is obtained for Pretoria from 2007 onwards.

## 4. Discussion

The aim of this study is to compare ground-based and satellite-derived UV index measurements at six South African sites. Two objectives were identified. When comparing the solar UV index measurements made by the ground-based stations versus the satellite-derived estimates, the correlation analyses showed that the two datasets were in good agreement with each other for most sites and time periods with an annual systematic bias (noted to be low at times). It is well known that satellite-derived solar UVR data have limitations, particularly for sites that are cloudy, polluted, and/or loaded with aerosols. In our study this was evident at the De Aar station. Additional data on factors influencing solar UVR are needed to compute the true ground-based solar UV index levels, to detail the comparison for sites with high bias, or improve the comparison for others. For example, if high resolution and quality cloud data were available at the ground-based stations, one could detect clear-sky days and compare clear-sky ground-based data with clear-sky satellite-derived data for solar UVR. However, cloud data are not routinely collected at the ground-based stations, hence, this subset of ground-based clear-sky solar UVR data cannot be determined.

Otherwise, we used the TUV model as another method of comparison. Ozone from the OMI satellite and the aerosol optical depth from MODIS were used as input parameters. The comparison between the UV index from the TUV model and the UV index from the OMI shows less than 5% of MAPE. The UV index from modelling does not provide more information for our study.

**Figure 10:**
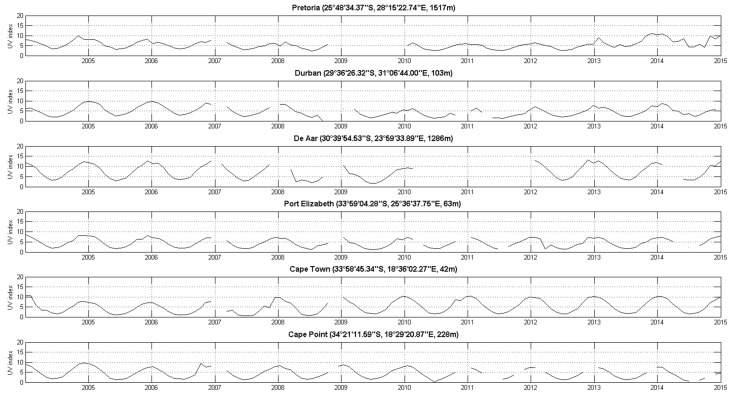
Ground-based monthly mean UV indices from 2004 to 2015 for the six South African sites.

The UV biometers are not manufactured with an intention of use for long-term trend analysis [29]. They are ideally suited to provide an indication of the UV index for public exposure assessment, risk awareness, and excess sun exposure prevention messaging. Hence, the intention here is not to detect long-term trends, but rather to compare our time-series to satellite observations and determine seasonal variability at different locations depending on longitude and altitude. However, there are subtle signs that the solar UV index levels at ground-based stations in South Africa have changed over the network measurement period. The raw data suggests that the intensity of the solar UV index is not consistent year-over-year; however, the pattern of change is irregular and it is impossible to make a definitive statement about any clear trend over time. This is shown by the monthly mean of the ground-based UV index (Figure 10) where one can identify an irregular pattern.

This relative difference may be explained by several factors including:

- OMI resolution can be a factor of this difference. Indeed, OMI data are integrated on a pixel of 1° × 1° (square of 110 km). In a region where cloud cover changes widely, it can quickly appear as a large difference with local data. A 1° × 1° pixel can also include a mountain and it is not taking into consideration the surface albedo, which may also have an effect [10,21,30,31]. This is evident at Cape Town and Cape Point stations. Ground-based observations show an evident difference, but this difference is transparent to the satellite.
- Ozone measured by OMI is an important factor of UVR variation. However, a recent study shows that the OMI satellite evaluates the total ozone at less than 5% accuracy in the South African region [32,33].
- Aerosols play an important part in the UVR response [30]. Global climatological aerosol datasets are used in OMI processing, but this is likely not relevant for an isolated, relatively clear site, like De Aar station, although wind-blown dust may be a factor.

We cannot prove which one, between ground-based and satellite datasets, is responsible for the large difference without additional high-grade solar UVR measurements, for example using Kipp and Zonen UVS-AB-T UV radiometers [28], being made alongside the UV biometers. Recently, the South African Weather Service, together with its partners, installed UVA/UVB radiometers at several sites as part of a solar radiation network. These instruments provide reliable data at high resolution and are strictly calibrated and maintained. It is anticipated that they will provide good quality data for future analyses.

## 5. Conclusions

In this paper we analysed ground-based UV indices recorded during the 1994–2015 period (more than two decades) by the SAWS (South African Weather Service) at six sites at different latitudes. In fact, South Africa has been measuring solar UVR to raise awareness about the impacts of the solar UV index on human health.

The SAWS UV index observations are based on the use of UV-biometers and the six datasets are not homogeneous in terms of time coverage and observations (Table 1). However, the present work is an exploratory study. It is based on the comparison with satellite UV index measurements from the OMI/AURA experiment. We found that relative UV index differences between ground-based and satellite-derived data range from 0 to 45%, depending on the site and year. Overall, there was a good agreement (absolute difference within ±25%) between South African ground-based station solar UV measurements and satellite-derived data from OMI/Aura, except for one station where an important bias is found. Most of the time, these differences appear in the winter, which emphasizes the importance of the annual cycle, in conjunction with altitude and latitude effects clearly underlined in our study on the UV index in South Africa.

Some of our ground-based stations’ data showed close agreement with satellite-derived UV index values. However, it should be noted that previous works showed that satellite-derived data can be overestimated by 11% [10], while free cloud filtering can also be improved by using different methods based on UV irradiance [15,34,35].

The ground-based instruments are not intended for long-term trend analysis; instead, they provide UV index information for public awareness, and some weak signs suggest such long-term trends may exist in our ground-based data. In future works we will study the variability and trends of the UV index in South Africa.

## Figures and Tables

**Table 1:** Geographical information of the six South African Weather Service ground-based UV index measurement sites.

Station	Geographical Position	Coordinates	Altitude	Time Series
Pretoria	1—FORUM Building	25.73° S, 28.18° E	1330 m	1994 to May 2003
	2—Erasmusrand	25.81° S, 28.49° E	1228 m	May 2003 to present
Durban	1—Louis Botha Airport	29.97° S, 31.00° E	9 m	1994 to May 2010
	2—King Shaka Airport	29.61° S, 31.11° E	103 m	May 2010 to present
De Aar	1—SAWS Building	30.67° S, 23.99° E	1286 m	2002 to present
Port Elisabeth	1—Port Elizabeth Airport	33.97° S, 25.61° E	63 m	2000 to present
Cape Town	1—Cape Town Intl Airport	33.98° S, 18.60° E	42 m	1994 to present
Cape Point	1—GAW station	34.35° S, 18.48° E	228 m	1997 to present

**Table 2:** Summary validation statistics for overpass UV index values (all available years) including bias, RMSE, and R^2^ values for the comparison of ground-based solar UVR and satellite-derived solar UVR (statistics computed only for clear sky day selected with LER).

Site	Number of Observation	Bias	MAPE	Median	SD ^#1^	RMSE	R^2^ Value	*p* Value ^#2^
	*n*	UVI	%	UVI	UVI	UVI	%	*p*
Pretoria	1578	−0.15	27.3	0.15	1.93	1.93	71.0	<0.001
Durban	1448	−0.59	28.8	0.59	1.48	1.57	88.5	<0.001
De Aar	1715	−1.73	46.5	1.67	1.85	2.57	87.9	<0.001
Port Elizabeth	1540	−0.19	23.1	0.34	1.46	1.46	84.9	<0.001
Cape Town	1827	−0.12	24.6	0.18	1.57	1.58	88.4	<0.001
Cape Point	1694	0.57	22.2	−0.28	1.51	1.62	87.4	<0.001

Note: ^#1^: Standard deviation; ^#2^: A *p*-value provides an indication of the level of statistical significance, *p* < 0.001 shows there is less than a 1 in a 1000 chance of the statistics computed in Table 2 being incorrect based on observed error.
